# Supplementary material for: Home birth and associated factors in Nigeria: A comparative study of rural and urban settings—Analysis of national population-based data
Source: PLoS One. 2025 Nov 21;20(11):e0337438. doi: 10.1371/journal.pone.0337438 (PMC12637914; doi:10.1371/journal.pone.0337438)
Supplement: Attachment — Submitted filename: Response to reviewers.docx [file pone.0337438.s002.docx]

**Authors’ response to reviewers’ comments**

Title: Home birth and associated factors in Nigeria: a comparative study of rural and urban settings—analysis of national population-based data (PONE-D-25-25157)

Authors:

Emmanuel O Adewuyi, Asa Auta, Olumuyiwa Omonaiye, Mary I Adewuyi, Victory Olutuase, Kazeem Adefemi, Olumide Odeyemi, Yun Zhao, Gizachew A Tessema, Gavin Pereira

Dear Editor,

We appreciate the meticulous review of our manuscript and the insightful comments and suggestions of the editor and reviewers. We have thoroughly considered these comments and revised our manuscript accordingly. Below, we present our point-by-point responses to the comments and suggestions. Line references refer to those in the tracked revised manuscript, except where otherwise noted.

In addition to the comments and recommendations of the reviewers:

1. We made a minor correction to the title, which avoids repetition of ‘based’.

New title:

**Home birth and associated factors in Nigeria: a comparative study of rural and urban settings—analysis of national population-based data**

2. We made minor corrections throughout to improve readability.
3. We proofread and spell-checked our manuscript.

**Editor Comments**

Journal Requirements:

Comment 1. Please ensure that your manuscript meets PLOS ONE's style requirements, including those for file naming. The PLOS ONE style templates can be found at

https://journals.plos.org/plosone/s/file?id=wjVg/PLOSOne_formatting_sample_main_body.pdf and

https://journals.plos.org/plosone/s/file?id=ba62/PLOSOne_formatting_sample_title_authors_affiliations.pdf.

Response: We thank the editor for the comment and guidance. We confirm that we have reviewed the links provided and have followed the templates from the outset (initial submission) of our manuscript. Additionally, we ensured that the naming of files complies with the requirements.

Comment 2. Thank you for stating in your Funding Statement:

“EOA was supported by the National Health and Medical Research Council (NHMRC; <https://www.nhmrc.gov.au/>) Investigator grants (GNT2025837). The funder has no role in the conduct of this study.” Please provide an amended statement that declares *all* the funding or sources of support (whether external or internal to your organization) received during this study, as detailed online in our guide for authors at <http://journals.plos.org/plosone/s/submit-now>.  Please also include the statement “There was no additional external funding received for this study.” in your updated Funding Statement. Please include your amended Funding Statement within your cover letter. We will change the online submission form on your behalf.

**Response:** We thank the editor for guidance regarding the Funding Statement, and have made the requested correction.

**Amended Funding Statement:** EOA was supported by the National Health and Medical Research Council (NHMRC; https://www.nhmrc.gov.au/) Investigator Grant (GNT2025837). The funder had no role in the conduct of this study. There was no additional external funding received for this study.

Comment 3. We note that you have indicated that there are restrictions to data sharing for this study. For studies involving human research participant data or other sensitive data, we encourage authors to share de-identified or anonymized data. However, when data cannot be publicly shared for ethical reasons, we allow authors to make their data sets available upon request. For information on unacceptable data access restrictions, please see http://journals.plos.org/plosone/s/data-availability#loc-unacceptable-data-access-restrictions.

Before we proceed with your manuscript, please address the following prompts:

a) If there are ethical or legal restrictions on sharing a de-identified data set, please explain them in detail (e.g., data contain potentially identifying or sensitive patient information, data are owned by a third-party organization, etc.) and who has imposed them (e.g., a Research Ethics Committee or Institutional Review Board, etc.). Please also provide contact information for a data access committee, ethics committee, or other institutional body to which data requests may be sent.

b) If there are no restrictions, please upload the minimal anonymized data set necessary to replicate your study findings to a stable, public repository and provide us with the relevant URLs, DOIs, or accession numbers. Please see http://www.bmj.com/content/340/bmj.c181.long for guidelines on how to de-identify and prepare clinical data for publication. For a list of recommended repositories, please see https://journals.plos.org/plosone/s/recommended-repositories. You also have the option of uploading the data as Supporting Information files, but we would recommend depositing data directly to a data repository if possible.

Please update your Data Availability statement in the submission form accordingly.

**Response:** Thank you for your guidance regarding the data availability statement. The data used in this study are from the Demographic and Health Surveys (DHS) Program. These data are owned and managed by the DHS Program, which imposes restrictions on their public sharing. As such, we are not permitted to upload or distribute the datasets directly. Access is granted by the DHS Program after registration and approval of a research request through their website (https://dhsprogram.com). Approval is determined by the DHS Program, which serves as the data custodian, to ensure that use complies with its ethical and legal requirements. Researchers wishing to access the data may submit a request to the DHS Program, which reviews applications and provides de-identified datasets under strict terms of use.

**Amended Data Availability Statement:** The data that support the findings of this study are available from the Demographic and Health Surveys (DHS) Program, but restrictions apply to their availability. These data were used under license for the current study and are not publicly shareable. Data are, however, freely available upon reasonable request from the DHS Program (https://dhsprogram.com/data/Access-Instructions.cfm) following their approval process. The authors did not have any special access privileges, and others may access the data in the same manner.

Comment 4. We note that Figure 1 in your submission contain [map/satellite] images which may be copyrighted. All PLOS content is published under the Creative Commons Attribution License (CC BY 4.0), which means that the manuscript, images, and Supporting Information files will be freely available online, and any third party is permitted to access, download, copy, distribute, and use these materials in any way, even commercially, with proper attribution. For these reasons, we cannot publish previously copyrighted maps or satellite images created using proprietary data, such as Google software (Google Maps, Street View, and Earth). For more information, see our copyright guidelines: http://journals.plos.org/plosone/s/licenses-and-copyright.

**Response:** We confirm that Figure 1 was entirely generated by the authors using R and open-source data. Specifically, the figure was created using the rnaturalearth and rnaturalearthdata packages to obtain publicly available shapefiles for Nigeria, along with ggplot2, sf, dplyr, and ggrepel for visualisation and labelling. No copyrighted maps, satellite imagery, or proprietary software (e.g., Google Maps, Google Earth) were used in generating this figure. As all components of the figure were created from public-domain data and original code, Figure 1 can be published freely without restriction.

To ensure transparency and reproducibility, we have made the R code used to generate this figure publicly available online at a GitHub page: ‘https://github.com/actright1/Nigeria-Geopolitical-Map’, which can be freely used by others.

Also, we have included a statement in-text**: ‘**Note: Map of Nigeria showing states and geopolitical zones. The map was generated by the authors using R with data from the rnaturalearth and rnaturalearthdata packages (public domain). A freely accessible script for generating the map is available at https://github.com/actright1/Nigeria-Geopolitical-Map (lines 233 - 236).

Comment 5. Please review your reference list to ensure that it is complete and correct. If you have cited papers that have been retracted, please include the rationale for doing so in the manuscript text, or remove these references and replace them with relevant current references. Any changes to the reference list should be mentioned in the rebuttal letter that accompanies your revised manuscript. If you need to cite a retracted article, indicate the article’s retracted status in the References list and also include a citation and full reference for the retraction notice.

Response: We have checked to ensure all references are complete and correct. To the best of our knowledge, there are no retracted studies cited in our work.

Additional Editor Comments :
**Comment:** This section could benefit with some critical appraisal
e.g Line 86-87; you mentioned the MSS and conditional cash transfers; however MSS was not sustained and conditional cash transfer was not widespread; provide a critical analysis of this and its probable contribution to differential outcomes across states/regions.

**Response:** We thank the editor for this suggestion. In the revised manuscript, we included an appraisal of both the MSS and CCT programmes. We also included the Basic Health Care Provision Fund (BHCPF).

*‘…While these programmes yielded some benefits, their overall implementation was limited. The MSS faced challenges with midwife retention, lack of ongoing training, inconsistent funding and lack of commitment from subnational governments [1, 2]. Similarly, CCT schemes were small-scale, intermittent, and in some cases withdrawn or delayed, undermining trust and continuity of care [3, 4]. The BHCPF, though promising in design [5-7], has struggled with underfunding, delays in fund disbursement, capacity gaps, weaknesses in supervision and coordination, and uneven rollout. These implementation challenges may partly explain the persistence of home births, a potential contributor to Nigeria’s high maternal and neonatal mortality.’ (101 – 108).*

**Comment:** line 97- home birth in Nigeria and LMICs; home birth in Nigeria is currently mostly TBA provided or unsupervised ‘SBA’, this is the core of your paper, hence will be useful to note this, and explore the whys of the contributions of these two ‘providers’ to poor maternal outcomes, including briefly touching why women will opt for a home birth based on past literature ( be careful when doing this in not giving too much away) as this is the focus of your research

**Response:** We clarified in the Introduction that most home births in Nigeria are attended by unskilled traditional birth attendants, while some occur without any attendant. We note that the focus of the present study is on estimating the prevalence of home births and examining associated factors across overall, rural and urban residences. Analysis of provider contributions to maternal outcomes is not the main focus. Brief contextual information (to focus on our topic and avoid making the writing too dense) from prior literature has been included to situate the study, without pre-empting the study findings, as provided in the paragraph below:

*‘…Yet in Nigeria, home births remain common [8-14], often attended by unskilled traditional birth attendants or, in some cases, without any attendant [15-17]. This premise may reflect both structural inequities and sociocultural practices, highlighting the need for a deeper understanding of the possible drivers of home birth in the country.’ (lines 119 – 122).*

**Comment:** line 161 –it will be useful to provide specific maternal health initiative that align with global health rather than just saying ‘some initiatives…

**Response:** We have revised the text to specify the maternal health initiatives implemented by the Nigerian government that align with global health priorities. We now mention the MSS and CCTs, which were designed to improve access to maternal and neonatal healthcare and service utilisation [1, 3, 4, 18].

*‘To improve maternal and neonatal health and align with global health goals, the Nigerian government has implemented initiatives such as the MSS, CCTs and BHCPF [1, 3-7, 18]. Despite these efforts, the country continues to experience poor maternal and neonatal health outcomes. Home birth is a potential contributor, and national data may obscure within-population disparities [19-21]; hence the approach in this study.’ (lines 266 – 271).*

**Discussion**
**Comment:** You mentioned using the theoretical frameworks Andersen’s Behavioural Model of Healthcare- this has not been integrated or applied explicitly in your discussion; will be beneficial to do this and not just limit to using it in identifying your explanatory variable. Consider how you can apply this to strengthen your discussion.

**Response:** We thank the editor for this valuable suggestion. We have now reflected Andersen’s model within the discussion. Beyond informing explanatory variables, we used the model’s domains: predisposing, enabling, and environmental factors, to briefly contextualise our findings. For instance, the region of residence, an environmental factor, was highlighted as potentially shaping the availability, accessibility, and utilisation of maternal health services, which underpins the observed regional and rural–urban disparities. Similarly, maternal and partner’s education, household wealth, ethnicity, parity, and media exposure were discussed as predisposing factors influencing mothers’ odds of home birth. Financial barriers, distance to facilities, and ANC utilisation were highlighted as enabling factors.

Together, these domains allowed us to interpret the observed patterns within a coherent theoretical framework. Importantly, we examined them not only through Andersen’s model but also through the broader lens of social determinants of health and social justice. This combined framing underscored how structural and systemic inequities may contribute to driving home birth in Nigeria, and highlighted the urgency of prioritising the most marginalised mothers in policy and practice. The approach was also reflected in our policy recommendations.

**Comment:** line 375-376- decline mentioned is from which data, kindly indicate what, and/or is it the previous DHS and why might this decrease have happened?

**Response:** We have clarified that the comparison figures are from the 2013 NDHS (with appropriate citation) and added a note that the modest decline may be due to the impact of interventions introduced in the past decades, while recognising that disparities persist and home birth remains prevalent. We prioritised being concise, so it does not become too dense, as we already have a lengthy discussion section.

*‘Although the figures represent a modest decline from the 2013 estimates (63% nationally, 78% rural, and 38% urban) [10, 14], the burden remains among the highest globally, with the national figure exceeding estimates in many African countries and the global average of 28% [22-25]. The modest decline observed in our study may reflect the impact of interventions implemented over the past decades in Nigeria [1, 3-7, 18]. (lines 547 – 552)*

*Despite these marginal improvements, the high burden of home births in Nigeria is concerning. The observed reductions fall short of global targets, measured by the proportion of childbirth attended by skilled personnel, a key indicator for achieving SDG 3.1 [26, 27]. The continued high prevalence is notable, as home births typically occur in Nigeria without access to emergency obstetric care or, in some cases, without anyone in attendance [15, 16].‘ (lines 556 – 562)*

**Comment:** Lines 380-382; similar to line 97-99 your introduction- possible repetition?

Response: We note that the introduction and discussion serve distinct purposes. In the introduction (lines 97–102 referenced from the previous submission), we present the general public health challenge of home births in LMICs and the associated risks. In the discussion (lines 380–382 of the previous submission), we applied the idea to our study findings by highlighting how the factors are associated in the Nigerian context (based on our findings). In the revised manuscript, we have double-checked to ensure everything harmonise well.

**Comment:** Line 384-385, explore further why urban residence alone does not guarantee for that region

**Response**: We have revised the discussion to concisely clarify why urban residence in the North-West may not necessarily guarantee a reduction in home birth. Specifically, we note that urban areas in this region may share many of the same systemic barriers as rural settings, such as poorly equipped facilities, insecurity, and entrenched cultural norms, which could limit effective access to maternal healthcare services. The remaining part of the paragraph provides further insight.

*‘…Even in urban areas, two-thirds (67%) of mothers in the urban North-West gave birth at home, showing that urban residence alone does not guarantee a reduction in the prevalence of home birth. This finding suggests that urban North-West share many of the systemic barriers with rural settings, including poorly equipped facilities, insecurity, and entrenched cultural norms [28]. (564 – 569).*

**Comment:** Line 389- what constrained options? This is too vague, kindly provide details/examples

**Response:** The ‘constrained options’ referred to are clarified in the sentence that followed the point where it was mentioned, which specifies systemic barriers such as inadequately equipped facilities, insecurity, and entrenched socio-cultural norms that limit access to skilled care in both rural and urban North-West Nigeria.

*‘The persistently high prevalence in areas such as rural North-West (89%) suggests that many mothers are compelled to give birth at home due to constrained options. These constrained options primarily reflect systemic barriers, including inadequate infrastructure, shortages of skilled personnel, insecurity, restrictive socio-cultural norms, and limited empowerment of women to make autonomous health decisions [28]. Critically, fewer than 20% of health facilities are equipped to provide emergency obstetric care [29], further restricting women’s choices.’ (lines 569 – 575)*

**Comment:** Line 396- you wrote “This premise also supports the urgent need for a social justice approach—one that goes beyond individual-level interventions to confront systemic barriers.”
Elaborate on what you mean by social justice approach in the context of your study

**Response:** We appreciate the comment. We have elaborated more on what we mean by the social justice approach in the context of our study.

*‘This premise also supports the urgent need for a social justice approach—one that goes beyond individual-level interventions to address systemic barriers by tackling the unequal distribution of resources, ensuring fair access to emergency obstetric services, and empowering women through education and autonomy [30-33]. In this context, a social justice approach means actively addressing structural inequities, such as poverty, gender disadvantage, and regional underdevelopment, that systematically marginalise disadvantaged mothers and constrain their ability to access safe childbirth care.’ (lines 582 – 588).*

**Comment:** Lines 436-439; you wrote “birth among women in the middle wealth category in urban settings only, possibly reflect the reality of urban poverty, where families live in informal settlements or slums, underserved by health infrastructure, or the erosion of the middle class in urban Nigeria”
This is too long, kindly split and rephrase to improve clarity.

**Response:** We have rewritten this section of the discussion and moved the text noted to the discussion for urban areas, where it more appropriately aligns. We ensured that the sentences are not too long.

*‘These findings suggest that economic barriers in urban Nigeria affect not only the poorest households but also the middle class, potentially reflecting rising costs, a shrinking middle class, and constrained access to affordable, quality maternity services [17, 34]. The finding may also reflect a growing form of urban poverty, highlighting widening urban inequities where socioeconomic disadvantage and spatial exclusion intersect. For instance, families in informal settlements or slums (due to low socioeconomic status) are disproportionately underserved by essential health infrastructure and often lack the resources to afford quality health care services [35].’ (lines 694 – 701)*

**Comment:** Lines 464-467- some critical analysis required in your suggestion on digital inclusion and integration of internet access into maternal health; will this not widen the existing health inequality, considering digital inclusion means access to smart phones- in the current climate of lack of basic infrastructure including electricity, poor internet connectivity, high cost of internet services; is this feasible across all states and all regions? Kindly note it is not just about suggesting policy that promote digital inclusion, what about the enabling factors to allow for the implementation of the policy, provide a crtiical discourse on this point.

**Response:** We acknowledge that promoting digital inclusion in maternal health could be challenging. Access to smartphones, reliable electricity, affordable internet services, and network connectivity is unevenly distributed across Nigeria, particularly in rural areas. We have made corrections and suggest that without addressing these enabling factors, policies promoting digital inclusion could inadvertently exacerbate existing inequalities rather than reduce them.

*‘We note that promoting digital inclusion in maternal health could be challenged by uneven access to smartphones, electricity, and network coverage, particularly in rural areas. Without addressing these barriers, digital interventions may widen existing inequalities [36]. Thus, policy efforts should combine digital inclusion with broader infrastructure improvements, including electricity, network expansion, subsidised internet, and community digital hubs.’ (lines 661 – 665)*

**Comment:** Line 475-477, you wrote “The markedly higher odds of home birth in the rural North-West and South-South may also exemplify how rurality and regional deprivation intersect to perpetuate maternal health disparities”. Kindly expand on what you mean – noting the keen differences in North and South of Nigeria Policy and programmatic implications.

**Response:** Thank you for the comment. We have expanded on the sentence to highlight that rurality could intersect with region-specific factors to shape maternal health outcomes. In the rural North-West, poverty, insecurity, and weak health-care infrastructure may increase the likelihood of home birth. In the rural South-South, geographic isolation, environmental degradation, and governance challenges could drive similar outcomes despite resource wealth. This premise underscores the need for context-specific interventions: strengthening health care and addressing insecurity in the North-West, and improving access, governance, and environmental resilience in the South-South. We have incorporated this explanation into the revised paragraph.

*‘The higher odds of home birth in the rural North-East, North-Central, North-West, and South-South reinforce the high prevalence observed in these regions, suggesting persistent geographical inequities. These disparities extend beyond mere regional differences; rurality potentially amplifies disadvantage through limited infrastructure, workforce shortages, and inadequate institutional presence. Within Andersen’s model [37], the findings implicate external environmental factors (region of residence) as shaping service availability, accessibility, and utilisation****.*** *The particularly elevated odds in the rural North-West and South-South further illustrate how rurality intersects with region-specific challenges to perpetuate maternal health disparities. In the North, these challenges could be intensified by poverty, insecurity, and weak health-care coverage [19, 38], while in the South-South, underinvestment persists despite resource wealth [19, 39]. Environmental degradation [40] and the geographic inaccessibility of riverine settlements further illustrates distinct forms of marginalisation [19, 39] in the South-South. These patterns indicate that the effects of rurality are not uniform across Nigeria, but are shaped by historical, political, and socio-economic contexts, including the uneven distribution of health facilities [19, 41].’ (lines 667 – 680)*

**Comment:** The way you have listed them all out in this section, your implications all appear like the typical suggestions. Going forward in rewriting this section; a critical consideration of feasibility will be beneficial in presenting this. Also, it is good for you to focus on innovative means (cost effective, community driven initiatives) that could be employed:
Consider the MSS that was started and abandoned due to number of reasons, including lack of collaboration from all partners. How can MSS be revamped and resuscitated? How do we address the user fee issue that limits access? What innovative community driven financial scheme can be used by women to address the use of user fee?
In considering the systemic/structural issues, what can you learn from the Abiye programme in Ondo state, why did it succeed and why has it gone down? What take home point can you use from this in addressing the systemic/structural issues
Still in line with above, controlling all other factors (like culture, ethnicity, or religion), the bottom line to the poor maternal outcome is the absence of good maternal health care system(systemic/strucutral factors) that meets the requirement of UHC (hence the need to look at the Abiye as mentioned above) what lessons can we learn and apply
Finally, since you used the social justice lens, kindly organise this section along that line so it does not appear like a ‘textbook prescription’,

**Response:** Thank you for the insightful comments. We appreciate the emphasis on feasibility, innovation, and the social justice framing. Our original list of policy and programmatic implications was based directly on the study findings and the rural–urban differences observed. However, we acknowledge that the current format may read as a conventional list of suggestions.

In revising this section, we have:

1. Considered the feasibility of each recommendation, including resource constraints and implementation challenges.
2. Highlight potentially innovative, community-driven approaches, such as collaborative financial schemes to address user fees and revamping existing initiatives like the MSS with active partner engagement.
3. Draw lessons from successful programmes like the Abiye program in Ondo State.
4. Organise the section around a social justice lens, emphasising equity, access, and the structural determinants of poor maternal outcomes.

We believe these revisions now strengthen the implications, making them more context-sensitive, actionable, and aligned with both feasibility and equity considerations. We have endeavoured to be concise in this sub-section, given that we have already had a lengthy discussion section. Please see the lines 811 – 890 for the details of the revised version of our study implication sub-section.

Minor corrections

**Comment:** Proofread and review for typo errors. E.g check your acknowledgement line 562, what is figs? Kindly refrain from the use of dash (-) in academic writing it sets an informal tone, and it is associated with AI generated work

**Response:** We have thoroughly proofread the manuscript and made all necessary corrections. The abbreviation ‘figs’ has been corrected to ‘Figures’. Concerning punctuation (dash), we confirm that we used em dashes (—) rather than hyphens/dashes, which are acceptable in scientific writing. Nevertheless, to avoid any perception of informality, we have carefully limited their use in the revised manuscript to ensure readability and maintain a professional style.

Reviewers' comments:

**Reviewer #1:**

**Comment 1**. Since you are using the Andersen model, kindly provide a brief description of the healthcare facility distribution in Nigeria (e.g., availability across states).

**Response:** We appreciate this comment. We have added a brief description of healthcare facility distribution in Nigeria, noting variations across regions and by rural-urban residences. Specifically, we highlighted that health facility availability is generally lower in northern and rural areas, while southern and urban regions have higher facility density. This information has been integrated into the Methods section as appropriate.

*‘Health facility distribution in Nigeria is highly uneven across states and regions. Of the 34,423 registered facilities, 88% are primary, 12% secondary, and only 0.25% tertiary centres, with about two-thirds publicly owned and one-third private [41]. On average, there are 16.6 primary, 2.2 secondary, and 0.05 tertiary facilities per 100,000 population; however, this figure masks sharp regional disparities. For example, the North Central has the highest density of primary facilities (23.3 per 100,000), while the South-South has the lowest (11.8 per 100,000). Secondary facilities are concentrated in the South-East (5.4 per 100,000), but remain scarce in the North-West (0.5 per 100,000). Tertiary facilities are clustered in the South-West and South-South, with a minimal presence in the North-East and North-West. Moreover, private facilities are more prevalent in southern states, whereas northern states such as Jigawa, Sokoto, and Zamfara rely more on public facilities [41].*

*‘Facility distribution also reflects a clear North–South and urban–rural divide: southern and urban areas host more private, secondary, and tertiary facilities, while northern and rural areas depend heavily on public primary centres, often under-resourced. These disparities are corroborated by the Lancet Nigeria Commission, which found that facility density is consistently higher in the south than in the north, and that several northern states also scored lowest on the health facility quality index, underscoring gaps in availability, infrastructure and staffing quality [19]. The uneven distribution of healthcare facilities in Nigeria may contribute to poorer access and lower quality of care in northern and rural regions. Within the Andersen model, these marked disparities in facility availability represent key enabling factors that influence service utilisation and help explain regional and socio-demographic differences in maternal health behaviours.’ (lines 246 – 262)*

**Comment:** 2. Line 185: Ethical approval was granted by the Nigerian National Health Research Ethics Committee, could you provide the ethical approval reference number from the Nigerian National Health Research Ethics Committee for this study?

**Response:** The approval we referenced here is not for our study, but the 2018 Nigeria Demographic and Health Survey (NDHS) protocol, which was reviewed and approved by the Nigerian National Health Research Ethics Committee (NHREC) and the ICF Institutional Review Board. To the best of our knowledge, the DHS documentation does not publish a unique NHREC approval reference number for the survey. However, the NHREC approval of the NDHS is explicitly noted in the official DHS methodology report [34]. Our study is based on a secondary analysis of public data, and no further ethics approval is required. We have revised the text to clarify this information (lines).

*‘Ethical approval for the NDHS 2018 survey was granted by the Nigerian National Health Research Ethics Committee, and informed consent was obtained from participants aged 18 and older, while parental consent was required for those under 18. The dataset can be accessed (https://dhsprogram.com/data/available-datasets.cfm) upon approval from the DHS Program [34]. As our study involved secondary analysis of fully de-identified publicly available data, no additional ethics approval was required.’ (lines 291 – 296)*

**Comment 3.** Table 1, the wording used for the enabling factors included was quite informal:
"Distance to health facility (big problem/not a big problem)"
"Permission to visit health facility (big problem/not a big problem)"
"Getting money for health services (big problem/not a big problem)".
Suggested revisions:
Difficulty due to distance (Yes/No)
Decision-making autonomy (Independent/Requires permission)
Affordability issue (Yes/No)

**Response:** The wording we used in Table 1 reflects the phrasing in the DHS questionnaire, where respondents were asked whether each factor (distance to facility, permission to seek care, and getting money for services) constitutes a ‘big problem’ or ‘not a big problem' in accessing health care. To remain consistent with DHS variable definitions and coding, we retained the original categories. However, we agree that a more concise and formal presentation could improve readability.

**Comment 4**. Table 2: Would you consider rearranging the order of the variables based on domain in Andersen model (to be similar to the order in Table 1)?

**Response:** Thanks for the comment. We have re-ordered the variables in Table 2 to follow the same approach in Table 1.

**Comment 5.** Table 2: Please consider adding a p-value column for clarity.

**Response:** We have included the p-value column in Table 2 as requested.

**Comment 6.** Line 454: Higher-parity women have higher odds of home birth; Line 502, Younger women have higher odds of home birth. However, younger women usually have lower parity, while higher-parity mothers are older. Could you reconcile or further explain these findings?

**Response:** We thank the reviewer for this valuable comment and for the detailed attention to the review. While age and parity could be related, they represent distinct predictors of home birth in our study. Our multivariable models adjust for both, allowing us to examine their independent associations. Importantly, our stratified analyses show they capture distinct influences. For instance, the association between higher parity and home birth was consistent across national, rural, and urban settings (Table 2), suggesting a robust effect whereby women with more childbirth experience are more likely to deliver at home. The finding is possibly due to increased confidence or reduced perceived need for skilled attendance. By contrast, the effect of younger age was significant only at the national and urban levels, but not in rural areas (Table 2). The urban influence was probably what was reflected nationally. This pattern indicates that younger mothers’ higher odds of home birth may be driven by context-specific barriers in urban settings, such as limited autonomy, financial dependence, or social constraints. Together, these results show that age and parity exert independent effects potentially through different mechanisms, and their influence varies by context. We have revised the Discussion to clarify this observation.

*‘Finally, it is noteworthy that our findings indicate both higher parity and younger age as predictors of home birth, which may seem contradictory. However, the results show that age and parity exert independent effects potentially through different mechanisms, with their influence varying by context (parity across rural and urban settings, age primarily in urban areas). These differences highlight the need for interventions tailored to the specific vulnerabilities of each group [37, 42]’ (lines 709 – 713)*

**Comment 7**. Line 500: You noted higher odds of home births among Muslim mothers. Could you provide supporting evidence on Muslim perspectives toward maternal health services in Nigeria, since you suggest culturally sensitive services?

Response: We have revised the manuscript to include supporting evidence and emphasised the need for culturally sensitive and faith-informed maternal health services to reduce home birth among Muslim women.

‘*Religion was also a significant factor in urban areas, with Muslim mothers being associated with higher odds of home births than Christians or followers of other religions. This finding may reflect cultural or religious considerations that influence maternal healthcare utilisation in Nigeria or correlations between religion and social determinants of healthcare access. Previous studies have reported that Muslim women faced barriers related to modesty, privacy, and preferences for female caregivers, which can limit their use of institutional maternal health services [43, 44]. These considerations underscore the importance of culturally sensitive and faith-informed maternal health services [45].*’ (lines 701 – 708)

**Reviewer #2:**

**Comment:** As far as i searched the study is original research and not published else where.
starting from the research topic it is impactful and pertinent issue but I have doubt on its novelty. since it was done before based on 2013 Nigeria demographic and health survey , it needs pertinent justification. even if conclusion lacks some reference data but recommendations were stated based on the finding data.

**Response:** We thank the reviewer for raising this point. While previous studies have examined home births in Nigeria using the 2013 DHS, our study is original in several ways. First, we used the most recent and comprehensive DHS data available, allowing for updated insights into home birth patterns in Nigeria. Second, our analysis disaggregates findings by national, rural, and urban settings and incorporates multiple socio-demographic factors. This detailed approach was not applied in the previous 2013 NDHS-based studies. Third, we contextualise our results through a social justice lens, providing actionable, context-specific policy recommendations that go beyond descriptive findings. These aspects establish the novelty and contribution of our research.

**Comment:** Better to select pertinent keywords in the abstract part

Response: We have refined the keywords to better reflect the central focus of our study, highlighting the main outcome, context, and methodological approach. The revised keywords are:

*‘Home birth, maternal and child health, rural–urban disparities, Nigeria, data disaggregation, inequalities, health facility delivery.’* (lines 76 – 78)

**Comment:** Introduction part is well written but significant impact of home birth not mentioned briefly add reference at the end of 1st paragraph of introduction part line number 87-89

Response: Thank you for the positive feedback and the observation. We have rewritten the introduction to ensure that home births feature more prominently, considering the focus of our study and feedback from other reviewers. In this revision, we have highlighted the significant impact of home birth on maternal and neonatal outcomes and added appropriate references at the end of the first paragraph. To provide appropriate context, we retain the first paragraph but brought in home birth earlier (within the first paragraph). We believe this section now reads more clearly and effectively reflects the importance of the issue. (lines 82 – 95)

**Comment:** Methods part how informed consent was applicable from secondary data? how do you get the participant? line number 185-187)

**Response:** We thank the reviewer for this point. Our study used secondary, de-identified data from nationally representative surveys, which were previously collected with informed consent from participants by the original survey teams. The reference to informed consent was in the section describing the data itself. We have double-checked and found there is no error in the description.

**Comment:** Operational definition should included in the method part to clarify some variables like rich house hold and poor house hold

**Response:** Thank you for this helpful suggestion. We have now included operational definitions for key variables in the Methods section, as necessary, to improve clarity and reproducibility (lines 320 – 342). The revisions, along with the information in Table 1, provide sufficient detail for readers to understand how each variable was measured and categorised.

**Comment:** Discussion part is well written and appreciated

**Response:** We appreciate the reviewer’s positive feedback.

**Reviewer #3:**

**General remarks about the manuscript
Comment:** This manuscript tackles a crucial public health issue—the ongoing challenge of home births in Nigeria and the disparities that exist between rural and urban regions. Utilizing a substantial, national dataset to examine these variations is a notable strength of the study. The emphasis on identifying related factors is particularly pertinent for shaping public health policies and interventions. The manuscript is well-articulated, and the research question is clearly defined. My following observations are aimed at enhancing the paper and preparing it for publication.

**Response**: We appreciate the reviewer’s positive feedback.

**Specific comments**

**Comment 1**. In result part of your abstract add confidence interval and p- value for all prevalence value or remove all, it is better to be similar. And also better to add p- Value and CI for the AOR value of significant variables and home birth.

**Response:** We have now included confidence intervals and p-values for all prevalence estimates in the abstract to ensure consistency. Regarding the AORs, we have summarised the results concisely in the abstract. Due to word count limitations, and to make our finding easily accessible, we did not include AORs in any of them for consistency. However, in the main manuscript, we have added a dedicated column for p-values alongside the AORs for all significant variables to provide complete statistical information.

**Comment 2.** Maternal morbidity and mortality, which are the results of home delivery, are discussed in the first paragraph of your introduction. But your first paragraph of introduction part is better to deal about home birth and the consequence of it in globally. Therefore, it is preferable to eliminate the first paragraph or reorganize the introduction's sequence.

**Response:** We thank the reviewer for this comment. We have substantially reorganised and improved the introduction, bringing home birth to the forefront of the background discussion while retaining the maternal mortality information, as these data provide important context for understanding the significance of home births. We believe the section now effectively highlights the global relevance and consequences of home birth while situating it within the broader maternal health context.

Comment 3. In your methodology part, the total 41,821 mothers were surveyed, but you take only 21,512 mothers why? Or how can you select those sampled mothers?

Response: The study sample of 21,512 mothers was derived from the total 41,821 surveyed in the 2018 NDHS. Not all mothers met the criteria for the current study. We included only mothers who had a live birth in the five years preceding the survey and for whom complete data on the place of childbirth for the most recent birth were available. Further, we restricted data to the most recent live birth per mother. This approach ensures a representative and reliable subset of respondents while maintaining the integrity of the statistical analysis.

**Comment 4.** In your analysis section, you assess the multicollinearity assumption, which is good, but what about other other logistic regression assumptions like outlier detection, the Hosmer and Lemeshow test, and the independence of observations?

**Response:** Thank you for this helpful comment. Given the complex nature of the survey data, we used SPSS Complex Samples Logistic Regression (CSLOGISTIC), which adjusts for stratification, clustering, and sampling weights. The Hosmer–Lemeshow test was not applied, as it is not supported in CSLOGISTIC and assumes simple random sampling, which is inappropriate for complex survey designs. Instead, model fit was assessed using Wald F-statistics, which are valid in this context. For instance, in the national model, the overall test was significant (F(36, 1278) = 67.511, p < 0.001), indicating strong model performance. We also examined pseudo-R-squared, and our analysis produced the following values for the overall population: Cox & Snell = 0.395, Nagelkerke = 0.533, and McFadden = 0.372. These values suggest a good to strong model fit for survey data.

Regarding the assumption of independence of observations, this was addressed through the complex sampling framework. We used the complex sample function of SPSS, specifying strata, primary sampling units (clusters), and sample weights in all analyses. This approach adjusts for potential intra-cluster correlations and ensures that standard errors and significance tests reflect the true variability in the population, thereby satisfying the independence assumption under the survey design. Further, to minimise potential statistical errors, we meticulously double-checked our analysis process to ensure all variables meeting the inclusion criteria were included in our models. Also, we rigorously validated the effectiveness of our backward elimination modelling by testing the final parsimonious models against potential confounding variables and factors previously reported to be associated with home birth. We have clarified these points in the revised manuscript (lines 357 – 373 in the methods section, and lines 435 – 454 for description of model fit in the results section). Thank you again for the valuable comment.

**Comment 5**. In you discussion part, the manuscript title highlights a comparative analysis between rural and urban settings. The discussion should not only present these differences but also delve into the potential underlying reasons for them. For instance, are the rural-urban disparities due to differences in healthcare infrastructure, socioeconomic status, cultural beliefs, or a combination of these factors? A deeper interpretation would significantly strengthen the findings.

**Response:** We thank the reviewer for this observation. Our discussion, particularly after this revision, now addresses the rural–urban disparities highlighted by the study. Specifically, we dedicated separate sections to interpreting findings in rural and urban contexts, linking them to systemic barriers, socioeconomic conditions, and cultural influences, and further tailored our policy recommendations accordingly. We believe the current version provides the deeper interpretation and extensive discussion that the reviewer suggested. We note the discussion is already extensive, and we endeavoured to be concise, so it does not become unnecessarily dense.

**Comment 6.** Ensure consistent use of terminology throughout the manuscript. For example, "home birth" and "childbirth outside health facilities" appear to be used interchangeably. A single, clear definition at the beginning of the manuscript would be beneficial.

**Response:** We have revised the manuscript to ensure that ‘home birth’ and ‘childbirth outside health facilities’ are not used interchangeably. We prioritised the term ‘home birth’ as this is the specific focus of our study. The few remaining instances of ‘childbirth outside health facilities’ occur mainly in contextual cases, such as when citing previous studies that used this terminology.

**Reviewer #4:**

Thank you for the opportunity to review this excellent and important paper. Overall, I think it is well done.

**Response:** We appreciate the positive feedback from the reviewer.

**Comment:** I do have some concerns that this paper was published with data from Nigeria without any authors who have an affiliation in Nigeria. I understand that maybe there are connections to Nigeria that I am not aware of but would like it to be addressed and explicitly stated if there is. If not, I would like to see this addressed in the limitations or potentially adding a reflexivity statement https://gh.bmj.com/content/9/1/e014743.

**Response:** We thank the reviewer for raising this point. While none of the authors currently hold institutional affiliations in Nigeria, 70% of the authors were born and educated in Nigeria. All these authors (from Nigeria) previously held appointments at one Nigerian institution or the other across the country. Additionally, the team maintains active collaborations and professional networks within Nigeria, which provide essential contextual understanding for the study. We trust that this clarification addresses the reviewer’s concern.

**Comment:** Line 81-specify "maternal and neonatal" mortality rates

**Response:** We have revised the text to explicitly specify both the maternal mortality ratio and the neonatal mortality rate, as suggested.

*‘According to the World Health Organisation (WHO), Nigeria’s maternal mortality ratio is 993 deaths per 100,000 live births (2023 data), classified as very high, and the highest globally [46, 47]. Similarly, the neonatal mortality rate, 34 per 1,000 live births (2023 World Bank data), ranks among the highest worldwide [48].’ (lines 90 – 93)*

**Comment:** Line 85-it is not just extremely high but the highest of any country in the world, I think this should be added.

Response: We thank the reviewer for this observation. We have corrected it to indicate that Nigeria has the highest number and maternal mortality ratio globally.

*‘Nigeria exemplifies this challenge: the country accounts for the highest number of global maternal deaths, over 28% of all deaths, and ranks high in the absolute number of neonatal deaths [46, 47, 49]. According to the World Health Organisation (WHO), Nigeria’s maternal mortality ratio is 993 deaths per 100,000 live births (2023 data), classified as very high, and the highest globally [46, 47].’ (lines 88 – 90)*

**Comment:** Line 88-Nonetheless, maternal and neonatal mortality remain unacceptably high in Nigeria, with the continued prevalence of home births emerging as a likely key contributing factor. -this should be referenced.

**Response:** We thank the reviewer for the comment. Yes, it should be referenced. We have rewritten the sentence for clarity, and we have now provided references as appropriate.

*‘Home births, often occurring without skilled attendance or timely access to emergency obstetric services, are a likely contributor to the unacceptably high mortalities [8, 10, 34, 42, 50, 51].’ (lines 93 – 95)*

**Comment:** line 99-lack of skilled birth attendants is also a significant factor here based on data and should be specifically called out.

**Response:** We have revised the sentence to highlight skilled birth attendants as a key factor. This idea now features well in our introduction section. See lines 93 – 95, where we introduce the concept (…*Home births, often occurring without skilled attendance or timely access to emergency obstetric services*). We provide other examples below:

*Lines 113 – 116:*

*‘In contrast, health facility births attended by skilled personnel improve outcomes through timely management of complications such as severe bleeding, infections, obstructed labour, and eclampsia—leading causes of maternal and neonatal mortality [8, 9, 52-54].’ (lines 113 – 116)*

*Lines 118 – 119:*

*‘Yet in Nigeria, home births remain common [8-14], often attended by unskilled traditional birth attendants or, in some cases, without any attendant [15-17].’*

**Comment:** line 101- Hence, ensuring facility-based births with access to emergency care remains essential to reducing these deaths. you should add and access to skilled birth attendants here.
I really love this dual approach -This dual lens shifts the focus beyond individual behaviours to systemic 137 constraints in maternal healthcare access.

**Response:** We have revised our introduction section. While the sentence here may have been completely rewritten, we ensure that the skilled birth attendants are reflected in all relevant places in the manuscript.

We appreciate that the reviewer likes the dual lens approach of social determinants of health and social justice approaches used in contextualising our findings.

**Comment:** The methods are sound and well done

**Response:** We appreciate the reviewer for this positive feedback on our methods.

**Comment:** Line 391-also women not be empowered also likely plays a role here and should be called out here as well as later when you discuss it more.

**Response:** We have revised the section to acknowledge that limited empowerment of women to make healthcare decisions is also a likely contributor to home births.

*‘These constrained options primarily reflect systemic barriers, including inadequate infrastructure, shortages of skilled personnel, insecurity, restrictive socio-cultural norms, and limited empowerment of women to make autonomous health decisions [28]’ (lines 571 – 574)*

Also, consistent with our findings, we had a further discussion (lines 680 – 689) and recommendations (858 – 865) related to the subject of women's empowerment and gender dynamics.

**Comment:** I would also add a line in the conclusions tying back in the maternal and newborn mortality to remind the readers why it is important to address home births.

**Response:** We thank the reviewer for the helpful suggestion. We have revised the conclusion to link the implications of home births to Nigeria’s persistently high maternal and newborn mortality. The revised conclusion now ends with:

*‘Addressing home births through such strategies represents an important step toward reducing Nigeria’s persistently high maternal and newborn mortality, underscoring the urgency of action.’ (lines 910 – 911)*

**Comment:** Very nice job with this paper

**Response:** We appreciate the reviewer for the positive feedback on our manuscript.

**Comment:** While revising your submission, please upload your figure files to the Preflight Analysis and Conversion Engine (PACE) digital diagnostic tool

**Response:** We have checked our figures using the PACE as requested.

**References**

1.	Abimbola, S., et al., *The Midwives Service Scheme in Nigeria.* PLOS Medicine, 2012. **9**(5): p. e1001211.

2.	Adegoke, A.A., et al., *Job satisfaction and retention of midwives in rural Nigeria.* Midwifery, 2015. **31**(10): p. 946-956.

3.	Okoli, U., et al., *Conditional cash transfer schemes in Nigeria: potential gains for maternal and child health service uptake in a national pilot programme.* BMC pregnancy and childbirth, 2014. **14**: p. 1-13.

4.	Ezenwaka, U., et al., *Influence of Conditional Cash Transfers on the Uptake of Maternal and Child Health Services in Nigeria: Insights From a Mixed-Methods Study.* Frontiers in Public Health, 2021. **9**.

5.	Chukwuma, J.N., *Implementing Health Policy in Nigeria: The Basic Health Care Provision Fund as a Catalyst for Achieving Universal Health Coverage?* Development and Change, 2023. **54**(6): p. 1480-1503.

6.	Igbokwe, U., et al., *Evaluating the implementation of the National Primary Health Care Development Agency (NPHCDA) gateway for the Basic Healthcare Provision Fund (BHCPF) across six Northern states in Nigeria.* BMC Health Services Research, 2024. **24**(1): p. 1404.

7.	Ibrahim, Z.A., et al., *Influence of Basic Health Care Provision Fund in improving primary Health Care in Kano state, a descriptive cross-sectional study.* BMC Health Services Research, 2023. **23**(1): p. 885.

8.	Chauke, L., *Improving access to emergency obstetric care in low- and middle-income countries.* Best Practice & Research Clinical Obstetrics & Gynaecology, 2025. **98**: p. 102572.

9.	Tura, G., M. Fantahun, and A. Worku, *The effect of health facility delivery on neonatal mortality: systematic review and meta-analysis.* BMC Pregnancy and Childbirth, 2013. **13**(1): p. 18.

10.	Adewuyi, E.O., et al., *Prevalence and factors associated with non-utilization of healthcare facility for childbirth in rural and urban Nigeria: Analysis of a national population-based survey.* Scand J Public Health, 2017. **45**(6): p. 675-682.

11.	Dahiru, T. and O.M. Oche, *Determinants of antenatal care, institutional delivery and postnatal care services utilization in Nigeria.* Pan African Medical Journal, 2015. **22**(1).

12.	Ononokpono, D.N. and C.O. Odimegwu, *Determinants of maternal health care utilization in Nigeria: a multilevel approach.* Pan African Medical Journal, 2014(ARTISSUE).

13.	Gage, A.J., O. Ilombu, and A.I. Akinyemi, *Service readiness, health facility management practices, and delivery care utilization in five states of Nigeria: a cross-sectional analysis.* BMC Pregnancy and Childbirth, 2016. **16**(1): p. 297.

14.	National Population Commission (NPC) [Nigeria] and ICF International, *Nigeria Demographic and Health Survey 2013*. 2014: Abuja, Nigeria and Rockville, Maryland, USA: NPC and ICF International.

15.	Fagbamigbe, A.F., et al., *Trend and decomposition analysis of risk factors of childbirths with no one present in Nigeria, 1990–2018.* BMJ Open, 2021. **11**(12): p. e054328.

16.	Austin, A., et al., *Trends in delivery with no one present in Nigeria between 2003 and 2013.* International journal of women's health, 2015: p. 345-356.

17.	Ntoimo, L.F.C., et al., *Why women utilize traditional rather than skilled birth attendants for maternity care in rural Nigeria: Implications for policies and programs.* Midwifery, 2022. **104**: p. 103158.

18.	Federal Ministry of Health. *Saving newborn lives in Nigeria: Newborn health in the context of the Integrated Maternal, Newborn and Child Health Strategy*. 2011; 2nd Edition:[Available from: https://dc.sourceafrica.net/documents/120214-NEWBORN-HEALTH-in-the-Context-of-the-Integrated.html.

19.	Abubakar, I., et al., *The Lancet Nigeria Commission: investing in health and the future of the nation.* The Lancet, 2022. **399**(10330): p. 1155-1200.

20.	Adewuyi, E.O., et al., *Antenatal care utilisation and receipt of its components in Nigeria: Assessing disparities between rural and urban areas—A nationwide population-based study.* PLOS ONE, 2024. **19**(7): p. e0307316.

21.	Adewuyi, E.O., et al., *Caesarean section and associated factors in Nigeria: assessing inequalities between rural and urban areas—insights from the Nigeria Demographic and Health Survey 2018.* BMC Pregnancy and Childbirth, 2024. **24**(1): p. 538.

22.	Appiah, F., et al., *Individual and community-level factors associated with home birth: a mixed effects regression analysis of 2017–2018 Benin demographic and health survey.* BMC Pregnancy and Childbirth, 2021. **21**(1): p. 547.

23.	Bado, A.R., et al., *Factors Associated With Home Births in Benin and Mali: Evidence From the Recent Demographic and Health Surveys.* Frontiers in Reproductive Health, 2022. **Volume 4 - 2022**.

24.	Dzomeku, V.M., et al., *Prevalence, progress, and social inequalities of home deliveries in Ghana from 2006 to 2018: insights from the multiple indicator cluster surveys.* BMC Pregnancy and Childbirth, 2021. **21**(1): p. 518.

25.	Hernández-Vásquez, A., H. Chacón-Torrico, and G. Bendezu-Quispe, *Prevalence of home birth among 880,345 women in 67 low- and middle-income countries: A meta-analysis of Demographic and Health Surveys.* SSM - Population Health, 2021. **16**: p. 100955.

26.	United Nations, *The Millennium Development Goals Report*. 2015, United Nations,: New York.

27.	World Health Organization, *World health statistics 2016: monitoring health for the SDGs, sustainable development goals*. 2016, WHO: Geneva.

28.	Geleto, A., et al., *Barriers to access and utilization of emergency obstetric care at health facilities in sub-Saharan Africa: a systematic review of literature.* Systematic Reviews, 2018. **7**(1): p. 183.

29.	Federal Ministry of Health Nigeria, *Second National Strategic Health Development Plan 2018-2022*. 2018, Federal Ministry of Health Nigeria Abuja, Nigeria.

30.	Marmot, M., *Social determinants of health inequalities.* The lancet, 2005. **365**(9464): p. 1099-1104.

31.	Macias-Konstantopoulos, W.L., et al., *Race, Healthcare, and Health Disparities: A Critical Review and Recommendations for Advancing Health Equity.* West J Emerg Med, 2023. **24**(5): p. 906-918.

32.	World Health Organization, *Operational framework for monitoring social determinants of health equity.* 2024: Geneva.

33.	Crear-Perry, J., et al., *Social and structural determinants of health inequities in maternal health.* Journal of women's health, 2021. **30**(2): p. 230-235.

34.	Nigeria Population Commission, *Nigeria demographic and health survey 2018*. 2019, NPC, ICF.

35.	Aliyu, A.A. and L. Amadu, *Urbanization, Cities, and Health: The Challenges to Nigeria – A Review.* Annals of African Medicine, 2017. **16**(4): p. 149-158.

36.	Badr, J., A. Motulsky, and J.-L. Denis, *Digital health technologies and inequalities: A scoping review of potential impacts and policy recommendations.* Health Policy, 2024. **146**: p. 105122.

37.	Andersen, R.M., *Revisiting the behavioral model and access to medical care: does it matter?* Journal of health and social behavior, 1995: p. 1-10.

38.	Olarewaju, O.A., *Insecurity in northern Nigeria: Implications for maternal and child health.* Clinical Epidemiology and Global Health, 2021. **12**: p. 100869.

39.	Onalu, C., et al., *Mortality Experiences for Women in Riverine Areas of the Niger Delta and Utilization of Maternal Health Services.* Health & Social Work, 2021. **46**(1): p. 59-67.

40.	Nkem, A.C., et al., *“Economic exclusion and the health and wellbeing impacts of the oil industry in the Niger Delta region: a qualitative study of Ogoni experiences”.* International Journal for Equity in Health, 2024. **23**(1): p. 183.

41.	Makinde, O.A., et al., *Distribution of health facilities in Nigeria: implications and options for universal health coverage.* The International journal of health planning and management, 2018. **33**(4): p. e1179-e1192.

42.	Adewuyi, E.O., et al., *Home childbirth among young mothers aged 15–24 years in Nigeria: a national population-based cross-sectional study.* BMJ open, 2019. **9**(9): p. e025494.

43.	Al-Mubarak, A., et al., *The Impact of Culture on Access to and Utilisation of Maternity Care Amongst Muslim Women in High-Income Countries: A Qualitative Systematic Review.* BJOG: An International Journal of Obstetrics & Gynaecology. **n/a**(n/a).

44.	Ganle, J.K., *Why Muslim women in Northern Ghana do not use skilled maternal healthcare services at health facilities: a qualitative study.* BMC International Health and Human Rights, 2015. **15**(1): p. 10.

45.	Solanke, B.L., et al., *Religion as a social determinant of maternal health care service utilisation in Nigeria.* African Population Studies, 2015. **29**(2).

46.	World Health Organization, *Trends in maternal mortality 2000 to 2020: estimates by WHO, UNICEF, UNFPA, World Bank Group and UNDESA/Population Division*. 2023: World Health Organization.

47.	UNICEF. *Maternal mortality*. 2025; Available from: https://data.unicef.org/topic/maternal-health/maternal-mortality/.

48.	The World Bank. *Mortality rate, neonatal (per 1,000 live births) - Nigeria*. 2023; Available from: https://data.worldbank.org/indicator/SH.DYN.NMRT?locations=NG&name_desc=false.

49.	World Health Organization. *Newborns: Improving Survival and Well-being*. 2020; Available from: https://www.who.int/news-room/fact-sheets/detail/newborns-reducing-mortality.

50.	Oyedele, O.K., *Disparities and barriers of health facility delivery following optimal and suboptimal pregnancy care in Nigeria: evidence of home births from cross-sectional surveys.* BMC Women's Health, 2023. **23**(1): p. 194.

51.	World Health Organization, *Standards for improving quality of maternal and newborn care in health facilities*, in *Standards for improving quality of maternal and newborn care in health facilities*. 2016.

52.	Musarandega, R., et al., *Causes of maternal mortality in Sub-Saharan Africa: A systematic review of studies published from 2015 to 2020.* J Glob Health, 2021. **11**: p. 04048.

53.	Say, L., et al., *Global causes of maternal death: a WHO systematic analysis.* The Lancet Global Health, 2014. **2**(6): p. e323-e333.

54.	Cresswell, J.A., et al., *Global and regional causes of maternal deaths 2009–20: a WHO systematic analysis.* The Lancet Global Health, 2025.
