# Supplementary material for: Home birth and associated factors in Nigeria: A comparative study of rural and urban settings—Analysis of national population-based data
Source: PLoS One. 2025 Nov 21;20(11):e0337438. doi: 10.1371/journal.pone.0337438 (PMC12637914; doi:10.1371/journal.pone.0337438)
Supplement: Attachment — Submitted filename: Response to Editor and Comments.docx [file pone.0337438.s003.docx]

**Response to Editor and Comments**

We appreciate the editor’s constructive comments and review of our manuscript. We have carefully considered each point and revised the manuscript accordingly. A detailed, point-by-point response is provided below. We have also taken this opportunity to conduct a thorough proofreading of the entire manuscript to enhance clarity and consistency.

**Comment 1:**
Repetition observed in lines 91–94 and 193–194. Consider merging the last paragraph under *Study Setting* with the paragraph above it and reframing lines 193–194 to read:
“Despite the Nigerian government’s efforts to improve maternal and neonatal health in line with global health goals, through initiatives such as the MSS, CCTs, and BHCPF mentioned earlier; poor maternal and neonatal health outcomes continue to persist.”

**Response:**
We appreciate this observation. The repetition has been addressed. The paragraph under *Study Setting* has been merged with the preceding one, and lines 193–194 have been revised following the suggested phrasing to improve coherence and eliminate redundancy.

*‘Despite the Nigerian government’s efforts to improve maternal and neonatal health in line with global health goals, through initiatives such as the MSS, CCTs, and BHCPF [1-7], mentioned earlier, poor maternal and neonatal health outcomes continue to persist.’ (lines 201 – 203)*

**Comment 2:**
Sentence in lines 235–236 should be in past tense.
“We adopt Andersen’s Behavioural Model as the conceptual framework and utilise the model in selecting explanatory variables.”

**Response:**
Thank you for this useful suggestion. The sentence has been revised to the past tense for consistency with the rest of the manuscript:

*‘We adopted Andersen’s Behavioural Model as the conceptual framework and utilised the model in selecting explanatory variables.’ (lines 244 – 245)*

Lastly, we have carried out a careful proofreading of the whole manuscript to enhance clarity and consistency.

**References**

1.	Chukwuma, J.N., *Implementing Health Policy in Nigeria: The Basic Health Care Provision Fund as a Catalyst for Achieving Universal Health Coverage?* Development and Change, 2023. **54**(6): p. 1480-1503.

2.	Igbokwe, U., et al., *Evaluating the implementation of the National Primary Health Care Development Agency (NPHCDA) gateway for the Basic Healthcare Provision Fund (BHCPF) across six Northern states in Nigeria.* BMC Health Services Research, 2024. **24**(1): p. 1404.

3.	Ibrahim, Z.A., et al., *Influence of Basic Health Care Provision Fund in improving primary Health Care in Kano state, a descriptive cross-sectional study.* BMC Health Services Research, 2023. **23**(1): p. 885.

4.	Abimbola, S., et al., *The Midwives Service Scheme in Nigeria.* PLOS Medicine, 2012. **9**(5): p. e1001211.

5.	Federal Ministry of Health. *Saving newborn lives in Nigeria: Newborn health in the context of the Integrated Maternal, Newborn and Child Health Strategy*. 2011; 2nd Edition:[Available from: https://dc.sourceafrica.net/documents/120214-NEWBORN-HEALTH-in-the-Context-of-the-Integrated.html.

6.	Okoli, U., et al., *Conditional cash transfer schemes in Nigeria: potential gains for maternal and child health service uptake in a national pilot programme.* BMC pregnancy and childbirth, 2014. **14**: p. 1-13.

7.	Ezenwaka, U., et al., *Influence of Conditional Cash Transfers on the Uptake of Maternal and Child Health Services in Nigeria: Insights From a Mixed-Methods Study.* Frontiers in Public Health, 2021. **9**.
